# Summing the strokes: energy economy in northern elephant seals during large-scale foraging migrations

**DOI:** 10.1186/s40462-015-0049-2

**Published:** 2015-09-15

**Authors:** JL Maresh, T. Adachi, A. Takahashi, Y. Naito, DE Crocker, M. Horning, TM Williams, DP Costa

**Affiliations:** Department of Ecology & Evolutionary Biology, University of California, Santa Cruz, USA; Department of Polar Science, Graduate University for Advanced Studies, Midoricho Tachikawa, Japan; National Institute of Polar Research, Midoricho Tachikawa, Japan; Department of Biology, Sonoma State University, Rohnert Park, USA; Department of Fisheries & Wildlife, Marine Mammal Institute, Oregon State University, Newport, USA; University of California Center for Ocean Health/Long Marine Lab, 100 Shaffer Rd., Santa Cruz, CA 95060 USA

**Keywords:** Accelerometer, Aerobic dive limit, Body size, Disturbance, Field metabolic rate, Foraging, Hypometabolism, Locomotion, Pregnancy

## Abstract

**Background:**

The energy requirements of free-ranging marine mammals are challenging to measure due to cryptic and far-ranging feeding habits, but are important to quantify given the potential impacts of high-level predators on ecosystems. Given their large body size and carnivorous lifestyle, we would predict that northern elephant seals (*Mirounga angustirostris*) have elevated field metabolic rates (FMRs) that require high prey intake rates, especially during pregnancy. Disturbance associated with climate change or human activity is predicted to further elevate energy requirements due to an increase in locomotor costs required to accommodate a reduction in prey or time available to forage. In this study, we determined the FMRs, total energy requirements, and energy budgets of adult, female northern elephant seals. We also examined the impact of increased locomotor costs on foraging success in this species.

**Results:**

Body size, time spent at sea and reproductive status strongly influenced FMR. During the short foraging migration, FMR averaged 90.1 (SE = 1.7) kJ kg^−1^d^−1^ – only 36 % greater than predicted basal metabolic rate. During the long migration, when seals were pregnant, FMRs averaged 69.4 (±3.0) kJ kg^−1^d^−1^ – values approaching those predicted to be necessary to support basal metabolism in mammals of this size. Low FMRs in pregnant seals were driven by hypometabolism coupled with a positive feedback loop between improving body condition and reduced flipper stroking frequency. In contrast, three additional seals carrying large, non-streamlined instrumentation saw a four-fold increase in energy partitioned toward locomotion, resulting in elevated FMRs and only half the mass gain of normally-swimming study animals.

**Conclusions:**

These results highlight the importance of keeping locomotion costs low for successful foraging in this species. In preparation for lactation and two fasting periods with high demands on energy reserves, migrating elephant seals utilize an economical foraging strategy whereby energy savings from reduced locomotion costs are shuttled towards somatic growth and fetal gestation. Remarkably, the energy requirements of this species, particularly during pregnancy, are 70–80 % lower than expected for mammalian carnivores, approaching or even falling below values predicted to be necessary to support basal metabolism in mammals of this size.

**Electronic supplementary material:**

The online version of this article (doi:10.1186/s40462-015-0049-2) contains supplementary material, which is available to authorized users.

## Background

Upper-trophic-level predators are important components of food webs, having disproportionate, landscape-level effects on the structure and function of ecosystems [1, 2]. Recent reductions in many species of large marine carnivores, including marine mammals, sharks and piscivorous fishes, have prompted calls for effective ecosystem-based management targeted at recovering depleted populations, while proactively protecting intact populations from decline [3–5]. As a result, many studies have focused on describing the distributions and foraging success of these groups in relation to habitat features [e.g., 6] and prey distributions [e.g., 7] with little information available on the basic resource needs of these species. Assessing the prey requirements of high-level predators is also central to determinations of how resilient they might be to ongoing anthropogenic disturbance and rapid environmental change [8]. In contrast to many terrestrial systems, information on energy requirements is difficult to come by for marine animals because they forage at sea, making their food habits and foraging behaviors challenging to directly measure.

In general, marine mammals have large energy requirements that are thought to be driven by the relatively large metabolic demands prescribed by carnivory [9] and the maintenance of a high core body temperature in water [10]. Foraging effort will reflect these requirements, and will contribute to energetic demands via the costs associated with locating, chasing and capturing prey [11]. To remain in positive energy balance, the energy acquired from foraging must exceed the energetic cost of foraging [12]. More successful foragers will accumulate surplus energy to allocate towards somatic growth and reproduction, and thus, a high foraging efficiency via the minimization of energy expenditure relative to energy gain is expected to be adaptive for all animals, and especially for predators with large energy requirements. Marine animals can minimize locomotion costs by adoption of stereotyped swimming behaviors. For example, “widely foraging” [13] individuals regularly engage in specific swimming modes [14], swim at particular speeds and depths [15], and utilize energy-saving swimming strategies like drift diving [16, 17], burst-and-glide swimming [18], porpoising [19] and wave-riding [20]. The disruption of these routine behaviors should increase the amount of time and energy spent foraging, resulting in increased locomotory costs and less energy devoted to production, thereby reducing foraging success.

**Fig. 1:**
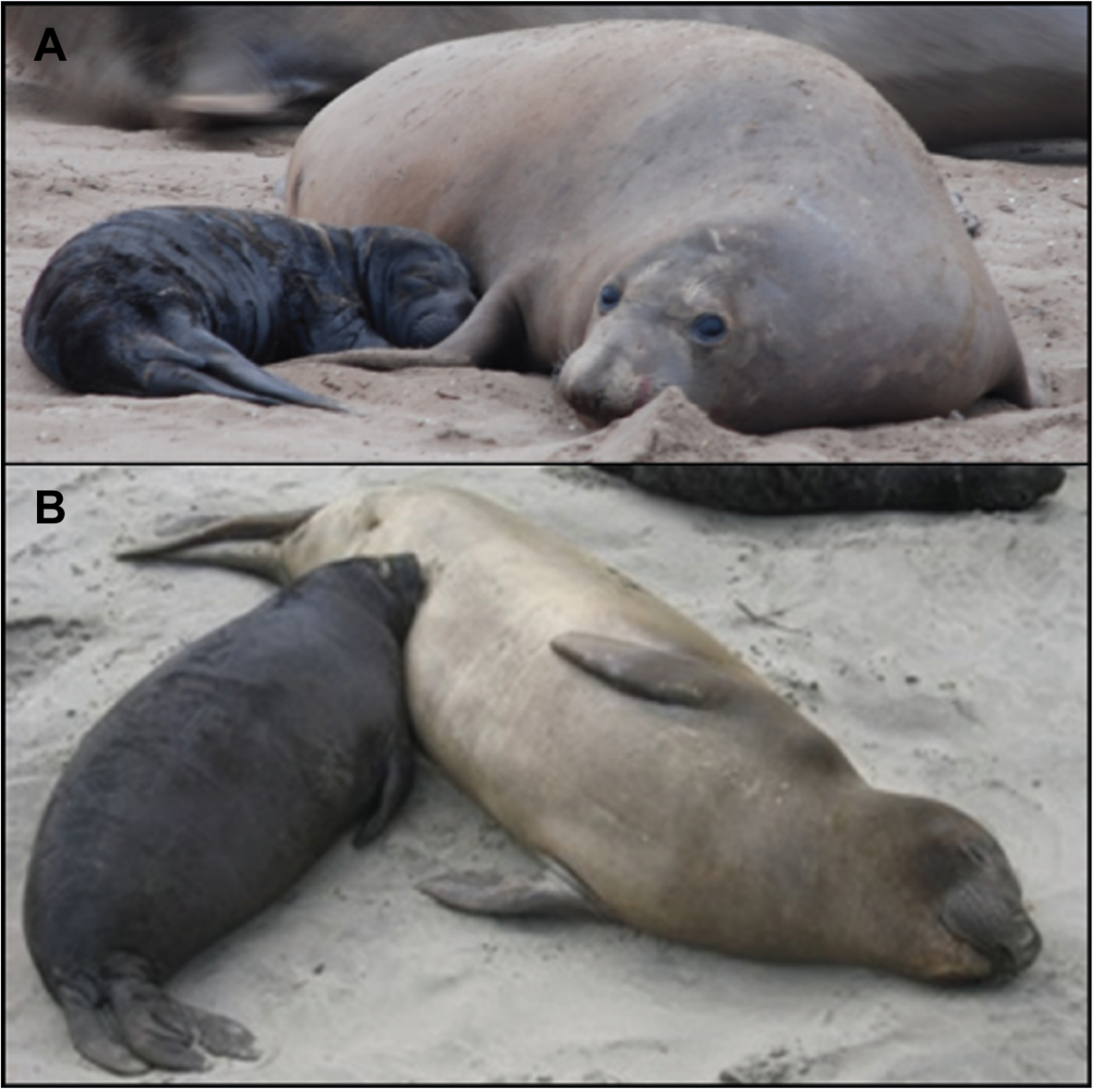
Northern elephant seal (*Mirounga angustirostris)* mother with **a** young pup (1–2 d) and **b** pup just before weaning (25–28 d). Photo credits: D. Costa, M. Fowler

The ecology of the northern elephant seal, *Mirounga angustirostris*, (Fig. 1) facilitates acquisition of foraging behavior data using archival tagging instrumentation, making it an ideal study species to address questions on the effects of both natural and anthropogenic disturbance on the foraging success of marine carnivores. Every year, adult females return to land for one month in between each of two foraging migrations, once to birth and nurse a pup, and once to molt their pelage [21]. Females are inseminated just prior to weaning, and then return to sea to forage for 2–2.5 months before hauling out for the molt. Implantation likely occurs during or after the molt, when seals return to sea for 7–8 months to forage and gestate the fetus. The demands for foraging success are considerable during this time, as pregnant seals must ingest sufficient energy to replace what was lost during the molt as well as store sufficient energy reserves to support the fasting mother and her suckling pup during the costly month-long lactation period [22].

Our objectives in this study were to determine the total energy requirements, and the relative partitioning of energy among competing demands, for adult female northern elephant seals under normal foraging conditions. We compare these data to those from 3 seals encountering increased foraging costs due to increased drag, to determine how the disruption of stereotyped locomotory behaviors affects energy balance. To achieve this, we constructed complete energy budgets by coupling measurements of foraging success (energy gain) during each foraging migration with empirical measurements of locomotion costs in free-swimming seals [23]. For one of the largest predators in the northern Pacific Ocean, we describe (1) increased, extreme energy economy as a function of pregnancy and/or time spent at sea; (2) strong, predictive relationships between body size, flipper stroking mechanics, and at-sea energy expenditure; and (3) reduced foraging success when stereotyped, energy-saving swimming behaviors are disrupted.

## Results

### Energy expenditure

Energy expenditure for each of the 22 seals carrying accelerometers is listed in Table 1. For each of the variables discussed below – field metabolic rates (FMRs), locomotion costs, and flipper stroking frequency – the response was influenced by the interaction between body size and foraging migration (short or long trip) (MLR results, *p* < 0.05 for each). The significant interaction term indicates that the relationship between mass and each response variable depends on the migration, so we ran MLRs on each of the migrations separately. As described below, our data indicate that, in general, locomotion behavior and the resulting field energetics of seals were most influenced by time spent at sea during the short trip, and the animal’s mass during the long trip. For both migrations, variance in calculations of FMR was most sensitive to estimates of CPS (Additional file 1: Table S.1); sensitivity of calculations of locomotion costs was spread among estimates of how ingested energy was partitioned among the input parameters E(Feces + Urine + Digestion + Maintenance) (Additional file 1: Table S.2).

**Table 1:** Summary of energy costs for northern elephant seals instrumented with accelerometers (*N* = 22)

Migration	Seal	Average Mass (kg)	DAS (TDR)	Stroke Rate (d^−1^)	FMR (kJ kg^−1^d^−1^)	Kleiber	Total E Spent (MJ)
ST: Short trip	U954	297	71.9	27840	90.7	1.28	1934
	U605	308	69.5	28548	91.1	1.30	1946
	U627	333	86.6	29972	97.4	1.42	2810
	T911	343	72.5	27423	85.8	1.26	2131
	1015	354	74.1	29100	92.9	1.37	2437
	X851	372	71.2	28674	94.6	1.42	2506
	T35	384	75.6	30385	96.6	1.46	2800
	N796A	391	72.8	28534	92.7	1.41	2640
	R541	392	77.7	29595	97.5	1.48	2973
	1733	406	78.2	27925	83.2	1.27	2642
	N796B	407	71.8	28818	92.4	1.42	2699
	W1095	433	79.5	29785	91.8	1.43	3163
	R382	466	69.3	24397	76.1	1.21	2459
LT: Long trip (pregnancy)	U458	341	222.1	22825	72.9	1.07	5519
	T730	343	218.8	23731	75.0	1.10	5623
	X106	379	230.8	21770	70.5	1.06	6170
	U754	395	221.1	23579	77.1	1.17	6727
	2036	445	224.5	19335	62.9	0.99	6287
	U203	450	224.5	18165	57.9	0.91	5841
DG: Short trip (added drag)	1234	374	100.8	34822	104.5	1.57	3932
	M780	378	78.1	34299	103.2	1.55	3044
	2370	433	76.6	35669	105.1	1.64	3484
Mean ST		376	75	28538	91.0	1.36	2549
(S.E.)		(13.5)	(1.3)	(422)	(1.7)	(0.02)	(103)
Mean LT		392	224	21568	69.4	1.05	6028
(S.E.)		(19.5)	(1.7)	(947)	(3.0)	(0.04)	(186)
Mean DG		395	85	34930	104.2	1.58	3487
(S.E.)		(19.1)	(7.8)	(399)	(0.6)	(0.03)	(256)

“Average Mass” is the seal’s mass averaged across the entire migration, based on her weight at the beginning and end of the trip. “Stroke Rate” refers to the number of flipper strokes per day averaged across the migration, and “FMR” refers to estimated field metabolic rates based on the total number of flipper strokes and a cost-per-stroke of 2.58 J kg^−1^[23] plus HIF costs. “FMR (Kleiber)” is a multiplier of Kleiber [43] predictions of mammalian basal metabolic rate. “Total E Spent” is the total amount of energy spent during foraging. See text for equations

#### Short trip

At-sea FMRs during the shorter migration averaged 91.0 ± 1.7 kJ kg^−1^ day^−1^, ranging from 1.2-1.5 times Kleiber predictions of BMR. There was a weak although not statistically significant predictive relationship between mass-specific FMR during the short trip, and mass and time spent at sea according to the equation:

1 $$ \mathrm{F}\mathrm{M}{\mathrm{R}}_{\mathrm{ST}} = 41.0\ {\mathrm{M}}^{-0.21}\ {t}^{0.51}\left({\mathrm{r}}^2 = 0.33,\ {\mathrm{F}}_{2,10} = 2.455,p = 0.14\right) $$

where FMR_ST_ is field metabolic rate during the short trip in kJ kg^−1^ d^−1^, M is mass in kg, and *t* is time spent at sea in days. Despite its low predictive (r^2^) value, eq. [2] was effective in estimating FMR_ST_ to within 6.3 ± 2.1 % of true values in the absence of flipper stroking data (Table 2).

**Table 2:** Comparison of different metrics for estimating at-sea FMRs of northern elephant seals

Migration	Seal	FMR (“True”) kJ kg^−1^ d^−1^	FMR (Alternate) kJ kg^−1^ d^−1^	FMR Error: (Alternate - “True“)
ST: Short trip	U954	90.7	93.6	3.03
	U605	91.1	91.2	0.14
	U627	97.4	99.8	2.42
	T911	85.8	91.2	5.90
	1015	92.9	91.4	−1.62
	X851	94.6	88.9	−6.48
	T35	96.6	90.8	−6.35
	N796A	92.7	87.2	−6.35
	R541	97.5	91.6	−6.42
	1733	83.2	91.2	8.78
	N796B	92.4	83.6	−10.52
	W1095	91.8	79.6	−15.34
	R382	76.1	83.5	8.79
LT: Long trip (pregnancy)	U458	72.9	76.3	4.42
	T730	75.0	76.0	1.28
	X106	70.5	70.5	−0.08
	U754	77.1	68.4	−12.71
	2036	62.9	62.6	−0.59
	U203	57.9	62.1	6.80
DG: Short trip (added drag)	1234	104.5	104.7	0.22
	M780	103.2	92.5	−11.52
	2370	105.1	83.3	−26.23
Mean ST		91.0	89.5	6.3
(S.E.)				(2.1)
Mean LT		69.4	69.3	4.3
(S.E.)				(2.8)
Mean DG		104.2	93.5	12.7
(S.E.)				(7.7)

FMR (“True”) is the field metabolic rate based on total number of measured flipper strokes and individual stroke costs, and FMR (Alternate) is based on the alternate metric with the strongest predictive relationship: mass and days at sea for ST and DG seals (eq. [2] in text), and mass for LT seals (eq. [4] in text). Error column represents percent difference in FMRs estimated using the different approaches, with mean (± SE) absolute error indicated

During the shorter migration, flipper stroking frequency was 28 538 ± 422 strokes d^−1^ and was best described by the equation:

2 $$ {\mathrm{R}}_{\mathrm{ST}} = 3\ 164\kern0.37em {t}^{0.51}\left({\mathrm{r}}^2 = 0.33,\ {\mathrm{F}}_{1,11} = 5.428,p = 0.04\right) $$

where R_ST_ is flipper stroke rate of actively-swimming seals during the short foraging trip in strokes d^−1^, and *t* is time spent at sea in days.

With basal costs [43] removed, the impact of each flipper stroke on locomotion costs was approximately 0.24 ± 0.04 J kg^−1^.

#### Long trip (pregnancy)

**Fig. 2:**
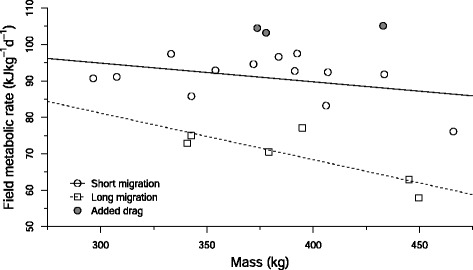
Mass-specific field metabolic rates of northern elephant seals (*Mirounga angustirostris)* based on total number of flipper strokes executed during their foraging migrations, as a function of mass. Filled circles indicate seals carrying added drag during their short migrations. See text for equations

**Fig. 3:**
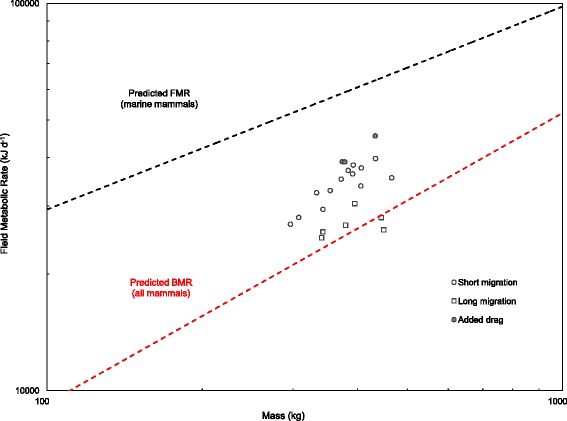
Field metabolic rates of northern elephant seals (*Mirounga angustirostris)* compared to Kleiber [43] predictions of mammalian basal metabolic rate (BMR) (dashed red line) and Boyd [12] predictions of marine mammal field metabolic rate (FMR) (dashed grey line), as a function of mass. Compared to seals during their short migration, seals of similar average body mass had 23 % lower FMRs during the long migration according to the equation FMR*_LT_ = 5818 M^0.26^

FMRs during the long trip (69.4 ± 3.0 kJ kg^−1^ day^−1^) were significantly lower than during the short trip (Welch two-sample *t*-test, t = 6.1972, df = 8.232, *p* < 0.001), therefore, we reject the null hypothesis that there is no difference in FMRs between migrations. Further, Cohen’s effect-size value and correlation (*d* = 3.2335, *r* = 0.8504) indicate a large effect with high practical significance and a strong correlation between migration and FMR. The largest seals expended the least amount of energy on a mass-specific basis during the longer foraging migration (Fig. 2), and compared to seals during the short migration, pregnant seals had lower mass-specific FMRs for a given size, falling below Kleiber predictions of mammalian basal metabolic rates (Table 1, Fig. 3). Mass-specific FMR in this group could best be described by the equation:

3 $$ \mathrm{F}\mathrm{M}{\mathrm{R}}_{\mathrm{LT}} = 5818\ {\mathrm{M}}^{-0.74}\left({\mathrm{r}}^2 = 0.65,\ {\mathrm{F}}_{1,4} = 7.568,p = 0.05\right) $$

where FMR_LT_ is field metabolic rate during the long trip in kJ kg^−1^ d^−1^, and M is mass in kg. Despite its low predictive (r^2^) value, eq. [4] was effective in estimating FMR_LT_ to within 4.3 ± 2.8 % of true values in the absence of flipper stroking data (Table 2).

Flipper stroking frequency was 24 % lower during the long migration (21 568 ± 947 strokes d^−1^) than during the short migration, and larger, pregnant seals stroked less (Table 1). Stroking frequency for seals during the long migration was best described by the equation:

4 $$ {\mathrm{R}}_{\mathrm{LT}} = 2\ 368\ 645\ {\mathrm{M}}^{-0.79}\left({\mathrm{r}}^2 = 0.68,\ {\mathrm{F}}_{1,4} = 11.56,p = 0.03\right) $$

where R_LT_ is flipper stroke rate of seals during the long foraging trip in strokes d^−1^, and M is mass in kg. We were unable to detect an effect of time spent at sea on FMR or flipper stroking frequency during the long trip.

With basal costs removed, the impact of each flipper stroke on locomotion costs was calculated as −0.50 ± 0.11 J kg^−1^, indicating an overestimation of basal costs in this group.

### Foraging success and energy budgets

**Fig. 4:**
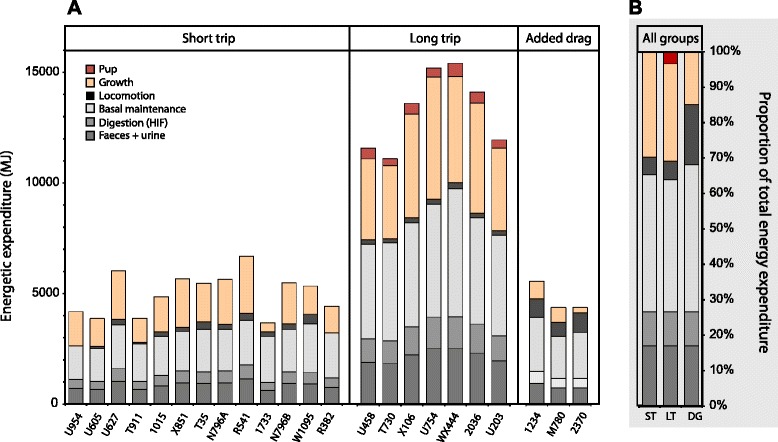
Partitioning of ingested energy among work costs (grey tones) and production (warm tones) in foraging elephant seals (*Mirounga angustirostris*). Absolute costs for each seal are shown in the white panels (**a**), while proportions of total costs are averaged across the three groups in the grey panel (**b**), where ST = seals during the short foraging trip, LT = seals during the long foraging trip, and DG = seals with added drag during the short trip. Within each group, seals are listed from left to right in order of increasing body size. If locomotion costs in LT seals are similar to those of ST seals, basal metabolism would have to be suppressed by approximately 22 % in pregnant seals (see text)

Mass gain, energy gain, and other indicators of foraging success for each seal are listed in Table 3. This information was used in combination with energy expenditures calculated above to determine the overall energy budget of each seal during her respective foraging migration (Fig. 4).

**Table 3:** Summary of mass and energy gains for elephant seals instrumented with accelerometers (*N* = 22)

Migration	Seal	Mass Gain (%)	Adipose Gain (kg)	Lean Gain (kg)	Net E Gain (MJ)	Gross E Gain (MJ)
ST: Short trip	U954	31.7	35.3	45.8	1539	4185
	U605	27.8	27.1	48.0	1266	3869
	U627	43.7	49.2	70.3	2187	6021
	T911	20.3	23.6	39.7	1086	3877
	1015	21.9	39.4	30.6	1589	4851
	X851	31.1	54.1	45.9	2205	5676
	T35	25.2	41.2	44.9	1742	5472
	N796A	29.5	48.6	52.0	2051	5652
	R541	37.2	61.9	61.1	2579	6688
	1733	11.8	4.4	40.8	413	3681
	N796B	26.9	42.8	53.7	1856	5488
	W1095	17.9	28.1	43.2	1268	5339
	R382	15.5	27.1	40.0	1215	4426
LT: Long trip (pregnancy)	U458	96.0	66.4	202.6	3635	11029
	T730	66.4	68.8	132.9	3280	10727
	X106	89.5	98.5	183.9	4655	13042
	U754	103.7	121.0	188.7	5480	14707
	2036	117.6	87.3	292.2	4944	13531
	U203	65.1	72.0	185.0	3721	11520
DG: Short trip (added drag)	1234	11.8	18.8	22.9	810	5714
	M780	8.9	16.7	15.5	690	4499
	2370	16.6	−0.4	41.7	252	4501
Mean ST		26.2	37.1	47.4	1615	5017
(S.E.)		(2.5)	(4.2)	(2.8)	(160)	(261)
Mean LT		89.7	85.7	197.6	4286	12426
(S.E.)		(8.5)	(8.7)	(21.3)	(354)	(645)
Mean DG		12.4	11.7	26.7	584	4905
(S.E.)		(2.2)	(6.1)	(7.8)	(170)	(404)

Mass Gain (%) is the increase in mass (post-migration) as a percentage of initial body mass (pre-migration). “Net E Gained” is the net energy gained during the foraging migration, and “Gross E Gained” is gross energy intake from prey before assimilation costs are deducted. For LT seals, all terms include mass or energy gained by both the mother and her gestating pup. See text for equations

#### Short trip

During the shorter migration, approximately 31.4 ± 2.1 % of total energy intake was allocated towards somatic mass gain (adipose + lean tissue), while 38.1 ± 7.2 % and 3.8 ± 0.1 % of total energy intake was devoted to basal metabolism and locomotion, respectively (Fig. 4). The remainder of total energy intake was lost as HIF and in the formation of urine and feces, as described in the methods.

#### Long trip (pregnancy)

During the longer migration, approximately 30.8 ± 1 % of total energy intake was allocated towards somatic mass gain (adipose + lean tissue), which was not significantly different from the shorter migration (GLS with fixed variance structure, F_2,16_ = −1.155, *p* = 0.88). Similarly, we were unable to detect an effect of mass on the proportion of total energy intake allocated towards somatic mass gain during either migration (GLS with fixed variance structure, F_2,16_ = −1.179, *p* = 0.26).

**Fig. 5:**
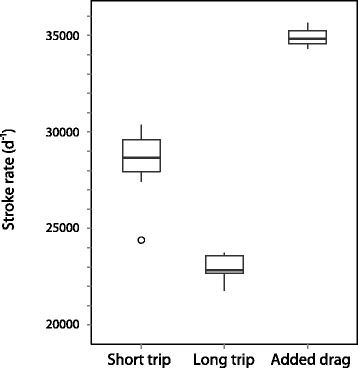
Flipper stroke rates were higher for northern elephant seals (*Mirounga angustirostris*) swimming normally during the short foraging trip (*N* = 13) than during the long foraging trip (*N* = 6). In comparison, seals swimming with added drag during their short trips (*N* = 3) stroked consistently faster than normally swimming seals during the same time (Welch two-sample *t*-test, t = −10.9982, df = 7.471, *p* < 0.001). Dark horizontal bars represent median (50^th^ percentile) values while the lower and upper limits of the boxes represent the 25^th^ and 75^th^ percentiles, respectively. Whiskers correspond to the 1.5 interquartile range, and points represent outliers

In contrast to somatic mass gain, the proportion of total energy intake utilized in locomotion was significantly lower (−7.7 ± 0.02 %) during the longer migration (GLS with fixed variance structure, F_2,16_ = 28.56, *p* < 0.001). We were unable to detect an effect of body mass on this proportion (F_2,16_ = 0.126, *p* = 0.64). Again, it is likely that locomotion results below zero are an artifact of inflated BMR estimates in this group. If we instead assume the energetic cost of each flipper stroke is the same for seals during both migrations, and because seals stroke 24 % less during the long migration (Fig. 5), we can estimate the actual proportion of total energy intake allocated towards locomotion in this group to be about 2.9 %. Fetal gestation costs consumed approximately 3.5 ± 0.3 % of total energy ingested during the long trip.

To “balance the budget” during the long migration, energy must have been shuttled away from metabolic overhead, which would require an approximately 22 ± 2.7 % reduction in BMR to achieve (Fig. 4). This reduction gives BMR a proportional contribution to overall costs that is comparable with that seen in the short-trip seals because mass-specific energy expenditure was lower in long-trip seals as described above. Thus, approximately 36.3 ± 1.1 % of total ingested energy was allocated towards BMR during the long trip instead of the approximately 47 % estimated before reduction in metabolic overhead was accounted for.

### Energy requirements

Seals in this study ingested an average of 5.02 × 10^3^ (±261) and 12.4 × 10^3^ (±645) MJ during the course of the short and long migrations, respectively (Fig. 4). For both groups, this ingestion rate is the equivalent of approximately 64–141 MJ per day spent foraging at sea (56 ± 2 and 126 ± 10 days during the short and long migrations, respectively), or 980–2200 kJ per foraging dive (3.74 × 10^3^ ± 161 and 8.36 × 10^3^ ± 581 total foraging dives during the short and long migrations, respectively).

### Seals with added drag

Seals swimming with added drag during the short migration (hereafter referred to as “drag seals,” *N* = 3) experienced a 14.5 % increase in FMR (Table 1, Fig. 2), resulting in half the mass gain of other short-trip seals swimming without the acoustic tags (hereafter referred to as “normally-swimming seals”) (Table 3). However, this should be interpreted as a conservative estimate of energy expenditure in the drag group as we have assumed stroking costs similar to those of normally-swimming seals. Two of the drag seals were at sea for as long as normally-swimming short-trip seals (74.6 days, S.D. = ± 4.8 days), but with substantially lower mass gain. The remaining drag seal (1234) spent 100.8 days at sea, with below normal mass gain results intermediate between those of the other two drag seals (Table 3).

Low mass gain in the drag seals resulted from the allocation of a disproportionately large amount of energy intake toward locomotion costs. Drag seals spent more than four times as much energy on locomotion (16.7 ± 0.02 % of total energy intake) as the normally-swimming, short-trip seals reported above, with a resultant one-third of the energy spent on somatic mass gain (11.7 ± 0.03 % of total energy intake) (Fig. 4). This was likely due to increased locomotion costs associated with overcoming the added hydrodynamic drag during diving and swimming, which is supported by the increased flipper stroking frequency demonstrated by the drag seals in comparison to normally-swimming, short-trip seals (22 % more strokes per day) (Fig. 5). The limited variation in stroking frequency for drag seals compared to the other groups suggests these individuals may have been pushing up against a biomechanically-constrained limit to swimming effort while foraging.

## Discussion

### Energy economy and the effects of pregnancy

The energy requirements of adult female northern elephant seals are much lower than those described for most other mammalian carnivores, and particularly so during pregnancy. Mammalian carnivores typically have higher energy needs than other terrestrial mammals, and thus require large food supplies to fuel fast metabolisms [9, 24]. As such, FMRs tend to run high in this group, ranging from 1.99 – 4.65 times Kleiber [43] predictions of BMR in terrestrial mammalian carnivores [summarized in 25, 26], and from 4.88–6.44 Kleiber predictions in marine mammal carnivores [27, 28] [although see 29, 30]. However, more recent studies on the diving metabolism (DMR) of adult phocid seals indicate they are more efficient than other marine carnivores [44, 33]. For example, Weddell seals diving in the wild for as long as the average elephant seal in our study (21.5 min) operated at 1.7 Kleiber [44].

During the 2.5-month post-breeding foraging trip, adult elephant seals were able to recover the energy reserves lost during lactation by operating at 1.36 (range = 1.21–1.48) Kleiber predictions of basal metabolism, indicating a high degree of metabolic efficiency in this species (Table 1, Fig. 3). These results are in line with results from previous studies on both free-swimming [23, 31] and captive [32–34, 28] elephant seals where large oxygen storage capacities combined with a high tolerance for hypoxia indicated these animals should be able to operate aerobically at 0.9–1.9 times Kleiber predictions from as early as 2 months old. While measurements in these examples were from young animals, recent studies on other phocids in captivity were unable to detect a difference between juvenile and adult DMRs [grey seals: 33] or BMRs [harp, harbor and ringed seals: 34]. As this could be an artifact of captivity, these results should be interpreted with caution; nonetheless, they do suggest that using measurements of metabolism in juveniles to ground truth our calculations for adults is a valid approach.

The degree of metabolic efficiency was correlated with body size and reproductive status, with the largest animals having the lowest mass-specific FMRs during pregnancy (Fig. 2). During the 7.5-month post-molt foraging trip, female seals were able to fuel gestation costs and a 90 % (S.D. = ± 21 %) increase in body mass by operating at FMRs approaching or falling below Kleiber predictions of basal metabolism (Fig. 3). In most mammals, pregnancy elevates metabolism [35, 36]; however, like other phocids, elephant seals fast during the breeding season and therefore must fuel lactation costs using onboard fuel reserves accumulated during the long foraging trip. Suppressed metabolism and increased fuel economy during pregnancy is likely a pre-pupping fattening strategy, and while it has been measured in resting, captive harp seals [31, 37, 34], grey seals [32], harbor and ringed seals [34], our study is the first to demonstrate suppressed metabolism during pregnancy in actively foraging, wild seals during their months-long migrations.

Our results provide empirical support for hypotheses that have inferred hypometabolism based on diving behavior, as elephant seals regularly dive for longer than their calculated aerobic dive limit [38] without engaging in a long recovery period afterward. These authors suggest that traditional predictions of diving metabolic rates based on allometric equations must be overestimates – instead, elephant seals must be hypometabolic while diving, and particularly so during the long foraging trip [39–42]. Compared to similarly-sized short-trip females,who are themselves operating at remarkably low metabolic rates (this study), long-trip seals were shown to suppress their field metabolism by a further 22 % (range = 15 % in smaller seals to 32 % in the largest), to rates below those predicted to be necessary to support even basic maintenance metabolism (Fig. 3). Boyd [12] predicted a similar trend for marine mammals using first principles, arguing that locomotion should be more efficient, and thermoregulation costs lower or non-existent, in larger aquatic animals.

The argument for a more severe degree of hypometabolism in northern elephant seals compared to other breath-hold divers is thus well-supported, and promotes the extreme at-sea lifestyle of this species. As part of the oxygen-conserving dive response, breath-hold triggers a reduction in metabolic rate in all mammals, and more markedly so in diving species [43, 44]. Extreme hypometabolism allows elephant seals to spend upwards of 95 % of their time at sea in breath-hold, exploiting depths down to 1600 m for up to 2 h, entirely aerobically. In contrast, other extreme divers such as Cuvier’s and Blainville’s beaked whales will regularly spend 60–90 min recovering between deep dives [45], suggesting that anaerobic metabolism is at least partly fuelling dives to extreme depths. These species have likely evolved adaptations that allow them to tolerate and process large amounts of lactic acid, whereas elephant seals seem to have evolved the ability to mostly just avoid it altogether with a more pronounced degree of hypometabolism. Other slow-moving, deep-diving marine mammals with short surface intervals, such as sperm whales [46], might also be expected to be hypometabolic.

**Fig. 6:**
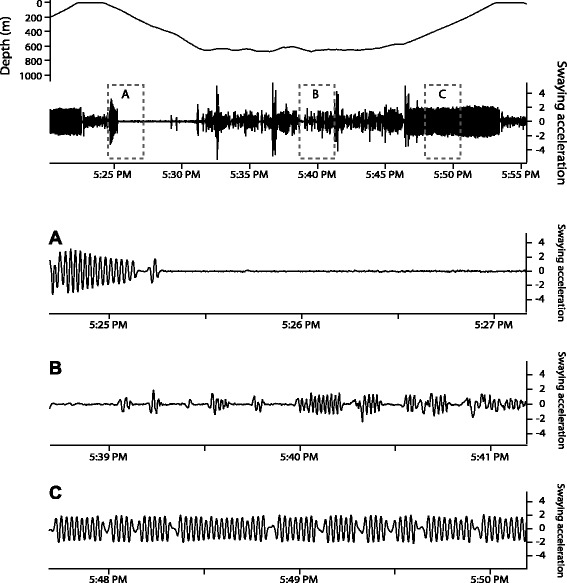
Flipper stroking of northern elephant seals (*Mirounga angustirostris*) follows a predictable pattern along the course of each dive. The top panel shows one foraging dive during the short migration of seal X851, where depth is shown with corresponding swaying acceleration. Grey boxes outline approximately 2.5-min segments of flipper stroking, each representing one of the three main phases of a dive cycle: (**a**) descent, (**b**) foraging at depth, and (**c**) ascent. Note the consistent, high-frequency flipper stroking occurring during ascent, when elephant seals are working against their negative buoyancy at depth in order to surface

In our study, lower at-sea FMRs in seals during pregnancy were also the result of reduced flipper stroking frequencies during active swimming (Fig. 3). For both groups, most flipper stroking occurs during the ascent phase of the dive cycle (Fig. 6), when seals must work against their negative buoyancy at depth to reach the surface [47, 48]. As the foraging migrations progress, seals are able to store more fat, becoming less negatively buoyant [16, 49], and we would expect an inverse relationship between buoyancy and the number of flipper strokes required to surface [50–52]. As seals generally gain more adipose tissue during the long trip (Table 3) a reduction in the number of flipper strokes necessary to surface compared to their short-trip counterparts is not surprising. With each flipper stroke having a predictable effect on overall energy costs, this reduced stroking frequency results in approximately 1700 ± 90 MJ in energy savings across the long foraging migration.

### Energy budgets

Despite the 5-month difference in trip duration, partitioning of ingested energy was remarkably similar across the two migrations. During the short trip, locomotion costs were low, constituting approximately 4 % of overall energy expenditure, which is somewhat lower than the 10 % reported for similarly-sized, freely-diving Weddell seals [calculated from eq.[3] in 44]. In contrast, locomotion costs of seals during the long migration were calculated as being negligible; however, this is likely the result of our assumption of an unvarying BMR equal to Kleiber predictions in these seals. As discussed above, there are several lines of evidence supporting the idea that ‘basal’ metabolism is less static in phocid seals than in terrestrial mammals, and that hypometabolism is common during pregnancy [31, 37, 32]. It is likely that this hypometabolism is the driver of reduced at-sea FMRs in pregnant elephant seal females rather than zero or negative locomotion costs. The physiological mechanism behind suppression is unclear, but may be a conditioning effect of chronic oxidative stress with increased time spent at sea in elephant seals [41, 53]. If locomotion costs on a per-stroke basis are instead assumed to be the same during both migrations, basal maintenance costs in pregnant elephant seals must be reduced by approximately 22 % (range = 14–33 %) of Kleiber predictions to “balance the budget” in terms of work costs (Fig. 4). This compares to a reported 30 % and 27 % reduction in the resting metabolism of captive, pregnant harp and grey seals, respectively [31, 32].

The overall net energy available to fuel production was highly variable for both migrations, with seals who were initially fatter tending to acquire more prey-energy, gain more weight, store more lipid, and birth larger pups (Table 3), even after controlling for maternal age. For example, seal 2036 increased her mass by 118 % compared to 66 % for T730, and gave birth to a 50-kg pup (versus 31 kg); however, 2036 was fatter than T730 at the beginning of her long migration, but had a lower total number of flipper strokes despite a longer trip duration (Table 1). We postulate a positive feedback loop whereby fatter seals are less negatively buoyant during the course of the trip, reducing locomotion costs as described above, freeing up more energy to devote towards both somatic growth (fattening) and fetal production. As adiposity increases and seals approach energetically-optimal buoyancy levels where locomotion costs are lowest [49], it stands that more energy would be available for allocation towards the growing fetus.

### Energy requirements

Seals in this study ingested an average of 5 017 (±261) and 12 426 (±645) MJ during the course of the short and long migrations, respectively (Fig. 4). Our estimate for short-trip seals is in complete agreement with Sakamoto et al.’s [54] estimate using an energy components analysis on the TDR record of one seal. Depending on the energy density of ingested prey items, we estimate that elephant seals in both groups would have needed to capture approximately 2-8 % of their average body mass in prey per day spent foraging, which is in close agreement with the 6.2 % predicted by Le Boeuf et al. [39]. This ingestion rate is the equivalent of 8–32 kg of prey captured per day spent foraging at sea which is, again, in agreement with Le Boeuf et al.’s [39] estimate of 20 kg based only on dive behavior. This ingestion rate is also the equivalent of 5–24 prey items per foraging dive, which is in agreement with Naito et al.’s [45] reported average of 14.6 (S.D. = ± 3) prey capture attempts per dive. Collectively, these lines of evidence support our stroke-based estimates of at-sea FMRs in adult female northern elephant seals.

### Disruption of routine swimming behaviors

The three seals carrying the experimental acoustic tags (“drag seals”) had FMRs elevated 14.5 % above other short-trip seals (Fig. 2), operating at approximately 1.58 (±0.03) Kleiber predictions of basal metabolism (Table 1). This was likely the result of increased locomotion costs, as the experimental tags increased the seal’s frontal surface area by about 7 %, which is large enough to induce increased transport costs in a number of large marine organisms [e.g., 55, 56]. Our results are comparable to what has been seen in other species such as Adelie penguins (*Pygoscelis adeliae*) where instruments representing an approximately 10 % increase in frontal surface area increased the cost of transport by about 14 % [57]. Increased locomotion costs are illustrated in Fig. 5, with drag seals flipper stroking consistently faster than normally-swimming short-trip and potentially pushing up against a biomechanically constrained maximum rate. With more energy partitioned toward the fuelling of flipper strokes (17 % versus 4 %), drag seals were able to partition relatively little ingested energy toward somatic mass gain (12 % versus 31 %) (Fig. 6).

These results suggest that elephant seals fuel their substantial mass gain during foraging migrations by engaging in stereotypic, energy-saving flipper stroking behaviors that keep locomotion costs low, and that increasing these costs can have considerable impacts on foraging success. This may have implications for the ability of elephant seals to adapt to at-sea disturbance, with avoidance behaviors predicted to reduce time spent foraging while increasing time spent in transit – a disruption of routine swimming behaviors that inflates normally-low locomotion costs, thereby reducing the surplus energy available to the seal for partitioning towards mass gain [as in, for example, 58, 59]. We predict that this effect would be exacerbated in pregnant, long-trip seals, which are potentially operating at or near a lower physiological limit to metabolism in order to adequately and rapidly build fuel reserves in support of a very costly lactation period.

## Conclusions

By accounting for each of the costs associated with foraging, we can assess the efforts free-living animals spend acquiring resources, and thus, their overall energy requirements [12, 63]. Although estimates of FMR have been made for fin [60] and minke whales [61], the seals in our study represent the largest carnivores measured empirically. Northern elephant seals have adopted a foraging strategy that utilizes a high degree of extreme energy economy, with FMRs that are (1) 70–80 % lower than predicted for carnivores of their size [52]; (2) 35–60 % lower than predicted for marine mammals of their size [12]; and (3) 25–40 % lower than what has been measured in freely-diving Weddell seals of similar size, and for similar diving durations [44]. Body mass was the most important determinant of FMR in our study, with a particularly dramatic effect of pregnancy such that, in the largest long-trip seals, Kleiber predictions of mammalian basal metabolism actually overestimated total at-sea energy expenditure. Pregnant seals were able to suppress their FMRs as body condition improved, thereby reducing the frequency of flipper strokes, and also by further reducing basal maintenance metabolism by an additional 22 % compared to non-pregnant seals during the short trip. The very efficient FMRs of seals during both the short (1.4x Kleiber) and long migrations (1.1x Kleiber) likely represent fattening strategies in preparation for the high energy demands of a month-long fast while molting and nursing, respectively. In contrast to normally-swimming seals, those instrumented with bulky, non-streamlined acoustic tags experienced elevated FMRs as a result of increased locomotion costs, significantly reducing foraging success and the net energy available for mass gain in these seals. Collectively, these results suggest that elephant seals keep overall energy requirements, and thus prey requirements, relatively low during their foraging migrations by engaging in adaptively stereotyped flipper stroking behaviors that minimize locomotion costs and, most likely, maintenance metabolism while diving. Minimization of these work costs frees up more of the energy ingested from prey items for fuelling of production, namely, accumulation of energy reserves for support of maintenance metabolism while fasting on land, and for pregnant seals, gestation and lactation.

## Methods

### Study animals

This project was approved by the Institutional Animal Care and Use Committee at the University of California in Santa Cruz. 21 adult female elephant seals were instrumented at their breeding colony in the Año Nuevo State Reserve, California, USA (37° 5’ N, 122° 16’ W) from 2009 – 2013. We chemically immobilized the seals for instrument attachment and recovery using established protocols that minimize handling time and stress [21]. Apparently healthy seals were selected and 15 were of known age ranging from 5 to 12 years old. N796 was instrumented in both 2009 and 2010, and we present each track separately. The study included both annual foraging migrations: the short, post-breeding migration (February through April; *N* = 16) and the long, post-molting, gestational migration (June through December; *N* = 7).

### Flipper stroking data

Seals were instrumented with a time-depth recorder (TDR) (Wildlife Computers MK9, MK10; or Lotek, St. John’s, NL, Canada: 2310) and a tri-axis accelerometer/magnetometer (Wildlife Computers MK10-style prototype, 16-hz sampling rate, *N* = 9, sample years 2009–2011; or Little Leonardo ORI2000-D3GT, 32-hz sampling rate, *N* = 13, sample years 2011–2013) for collection of at-sea diving and flipper stroking data, respectively. The raw time-series of accelerometry measurements were truncated according to departure/arrival times identified using the diving record, and flipper strokes isolated using one of two custom-written programs in Igor Pro 6.36 (WaveMetrics, Inc., USA), depending on the instrument model used. In brief, side-to-side flipper movements were detected as fluctuations in the transverse axis – “swaying” acceleration – and the static (positional) component was separated from the dynamic (movement) component using a 1 Hz low-pass filter [62]. The remaining peaks and troughs in the dynamic swaying acceleration with amplitudes greater than 1 m s^−2^ were considered to be individual flipper strokes and were used in analyses.

Output from the Wildlife Computer instruments included raw acceleration data, and a user-written algorithm was used to identify and count individual flipper strokes [23]. In contrast, with the exception of 12 h per record, raw data were processed on-board the Little Leonardo instruments with stroke rate calculated using a built-in algorithm. To make comparisons between algorithms, we processed each seal’s 12 h of raw Little Leonardo accelerometry data through our user-written algorithm and used the percent discrepancy between stroke counts to correct the total number of counts output by the Little Leonardo algorithm. In most cases, the total number of strokes counted by the two algorithms were within 10 % of each other; however, in 2 cases, the discrepancy was greater than 10 % (14 % for T35, 20 % for T730). For this reason, and for consistency, we used corrected counts from the Little Leonardo instruments, rather than the processed output from the instruments, in analyses.

As part of a separate study, three seals were additionally outfitted with prototype acoustic tags, for testing of their viability in future studies. The tags were large, not streamlined, heavy, and were as follows: cylindrical tags (537.0 x 117.4 mm, cross-sectional area 108.3 cm^2^, volume 5447 ml, mass in air 7500 g, mass in water 2417 g) attached along the midline of the seal’s back, with the forward leading edge at the position of the seals’ maximum girth. The tags were attached using two positively buoyant foam block mounts (each cross-sectional area 28.2 cm^2^, mass in water 1151 g), hose clamp screw (cross-sectional area 2 cm^2^), and VHF transmitter (cross-sectional area 7.7 cm^2^) for a total cross-sectional area of approximately 174.2 cm^2^ that corresponds to approximately 7 % of the seals’ cross-sectional area (ca. 2501.9 cm^2^), and mass in water of 1266 g.

The tags were not deployed with the intention of affecting the foraging success of the animals, but upon recovery these seals were undersized and clearly nutritionally stressed, probably due to the added hydrodynamic drag imposed on the animals by the bulky, non-streamlined instruments. We include these individuals in our analyses here to determine how foraging success and efficiency are affected by disturbance to routine swimming behaviors via increased locomotory costs.

### Energetics data

The surplus energy available to a seal for production of new tissue is a function of the difference between gross food energy ingested at sea and energy lost or expended while foraging. In our study animals, production of new tissue refers to the replenishment of spent energy reserves as both adipose and lean tissue gain, and the gestation of a fetus. Production is therefore defined here as the total mass gained from somatic and fetal growth, and can be described by the equation:

5 $$ {\mathrm{E}}_{\mathrm{MASSGAIN}\ \left(\mathrm{somatic} + \mathrm{fetal}\right)} = {\mathrm{E}}_{\mathrm{INGESTED}}\hbox{--}\ \mathrm{E}\left(\mathrm{Feces} + \mathrm{Urine} + \mathrm{Digestion} + \mathrm{Maintenance} + \mathrm{Locomotion}\right) $$

where some energy from ingested prey items is lost in the production and excretion of feces and urine, and some energy is expended to fuel digestion costs [63, 64], basal maintenance metabolism and locomotion. Collectively, energy expended for digestion, maintenance and locomotion represent the animal’s field metabolic rate (FMR). We do not include heat lost for thermoregulation as a cost as it has been argued [12, 65] and demonstrated [66] that heat loss should not be an issue for marine mammals of this size.

To estimate surplus energy gained from foraging (E_MASSGAIN (somatic + fetal)_), we measured the mass of each seal at the beginning and end of each trip by suspension in a canvas sling from a tripod using a Dyna-Link scale (1,000 ± 1 kg). Mass of adult females at departure and upon arrival was corrected for any time spent on land after instrument attachment or before instrument retrieval, respectively, using an equation derived from serial mass measurements of fasting seals [67]. During the breeding season, when female seals return from their long, post-molt, gestational migrations, the mass of the pup was added to that of the mother five days post-parturition. Waiting a conservative 5 days to handle the newborn pup is part of standard protocol with this species, in order to allow adequate time for maternal contact and bonding (e.g., [68]). Adipose and lean tissue gain was estimated from mass change and body composition, assuming that the five-day-old pup was 13 % adipose tissue [22]. Energy gain was estimated assuming that adipose tissue was 90 % lipid, and lean tissue was 27 % protein with a gross energy content of 39.33 kJ g^−1^ for lipids and 23.5 kJ g^−1^ for protein [22]. Additional gestation costs associated with maternal metabolism were assumed to be negligible based on previous research on other capitally breeding phocids [69, 70], and so were not added to the energy budget of seals in this group.

To estimate energy expenditure (FMR) during the foraging migrations, we used an equation that predicts total FMR from the total number of swim strokes in free-swimming, non-reproductive, fasting seals [FMR (J kg^−1^) = 2.58Sn, where Sn is the number of flipper strokes [23]]. This equation was derived from seals younger than the ones in this study, requiring us to rely on the assumption that juvenile and adult animals have similar stroke costs. This might be unrealistic as juvenile mammals often have elevated mass-specific metabolisms relative to adults as a result of increased growth costs. If this were the case, our FMR estimates for adults would be too high. However, Maresh et al. [23] were unable to detect significant relationships between body size or age and stroke costs, although this could have been due to small sample size and lack of statistical power. On the other hand, multiple studies have been unable to detect differences between juvenile and adult metabolic rates in captive phocids [71, 72], indicating that our assumption of similar stroking costs in juvenile and adult elephant seals is reasonable. We consider the sensitivity of our FMR calculations to uncertainty in stroke costs in the Additional file 1.

As FMR represents the sum of all component costs except E_feces_ and E_urine_, (i.e., FMR = E(Digestion + Maintenance + Locomotion) from eq.[1]), we estimated each separate cost and its relative contribution to total FMR using values and equations from previous studies (described below). To account for energy lost in via feces and urine, we used a value averaged across multiple studies on phocid seals [63, 73, 74] whereby approximately 83 % of gross energy consumed from an average fish diet is usable as metabolizable energy. This value for assimilation efficiency is in line with studies on other pinnipeds where metabolizable energy was shown to range from between 78.3–91.6 %, depending on the diet [75]. To account for digestion costs (the heat increment of feeding (HIF)), we used the estimate of 11.6 % of metabolizable energy measured in juvenile elephant seals (range = 6.4–18 %) [76]. This value is in close agreement with other studies of HIF in marine mammals: 10–13 % in sea otters [77], 10–17 % in harp seals [78, 79], and 5.5 % in harbor seals [80].

Basal metabolic rate (BMR) has not been measured for adult female elephant seals, however, BMR values predicted from Kleiber [81] have been shown to be within 10 % of the metabolic rates of quiescent, submerged Weddell seals [82]. In addition, Lavigne et al. [63] found no difference between Kleiber’s predicted BMRs of terrestrial mammals and the empirically-determined BMRs of similarly-sized, adult phocid seals when measured under similar conditions. For these reasons, we used Kleiber’s predictions of BMR for terrestrial mammals to estimate the maintenance costs of the seals in this study.

Finally, after accounting for assimilation efficiency, HIF and BMR, any remaining cost was assumed to represent energy spent on locomotion.

With all costs and gains accounted for, we could then estimate the energy ingested from prey that was necessary to balance each seal’s energy budget (E_INGESTED_). We estimated the energy from prey our study animals would have needed to consume overall as well as on a per foraging dive basis. Foraging dives were determined using a custom-written dive typing script in MATLAB (IKNOS toolbox, Y. Tremblay, unpublished program), whereby the putative behavior of the seal is classified based on the two-dimensional shape of each dive as recorded by the TDR [30]. In addition to the sawtooth-shaped dives traditionally classified as foraging, we included the V-shaped dives traditionally classified as transit, per recent evidence provided by Naito et al. [83] that demonstrates the high probability that northern elephant seals are foraging during these dives as well.

### Statistical analysis

The influence of body size, time at sea and foraging migration (short or long trip) on field metabolic rate, locomotion costs and flipper stroking frequency, was investigated using multiple linear regression (MLR) models. Candidate models included the interaction term body size x migration to test whether the effect of body size on each of the response variables co-varied with migration, in which case we ran MLR models on each of the migrations separately. These MLRs included time at sea and its potential interaction with body size. Generalized least squares (GLS) models with variance structure fixed for mass were used to measure the association between body size and foraging migration and the proportion of ingested energy allocated towards mass gain and towards locomotion costs. All means are expressed as ± S.E. of the mean, except where noted otherwise. Analyses were performed using the built-in ‘lm’ function, and the ‘gls’ function of the ‘nlme’ package in R 3.1.2 [84]. All model combinations were fitted with best model fits based on the lowest Akaike information criteria corrected for small sample size (AICc). Where *p*-values indicate significant differences (*p* < 0.05) between seals in the two groups, we report Cohen’s *d* effect sizes and effect-size correlation r_γλ_ [85, 86], using the long foraging migration (when seals were pregnant) as the ‘treatment’ effect. In addition, we analyzed the sensitivity of FMR and locomotion cost model results to uncertainty in the estimates of input parameters using a Latin hypercube random sampling method that takes into account the range and distribution of each input parameter, as well as their interactions [87]. Sensitivity analyses were performed using the ‘sensitivity’ , ‘pse’ , ‘ks’ and ‘Hmisc’ packages in R and the Excel ‘Apogee’ add-in v.4.9 developed by the Statistical Design Institute (Additional file 1).

## Data accessibility

All data used are present in the manuscript and its supporting information.

## Additional file

Additional file 1: Table S1. Sensitivity results for calculations of field metabolic rate (FMR, in kJ kg^-1^ day^-1^) in adult, female northern elephant seals (*Mirounga angustirostris*) during the short and long foraging migrations. **Table S2.** Sensitivity results for calculations of locomotion costs (%Locom, as a percentage of ingested prey energy used) in adult, female northern elephant seals (*Mirounga angustirostris*) during the short and long foraging migrations. (DOCX 18 kb)

## Abbreviations

ADL: Aerobic dive limit
cADL: Calculated aerobic dive limit
BMR: Basal metabolic rate (kJ kg^−1^ d^−1^)
DAS: Time spent at sea (d^−1^)
DG: Drag treatment; refers to seals carrying added drag during the short foraging migration
DMR: Diving metabolic rate (kJ kg^−1^ d^−1^)
FMR: Mass-specific field metabolic rate (kJ kg^−1^ d^−1^)
FMR*: Absolute field metabolic rate (kJ d^−1^)
HIF: Heat increment of feeding
LT: Long trip; refers to seals during the long foraging migration (pregnant)
R: Stroke rate (strokes d^−1^)
Sn: Total number of strokes
ST: Short trip; refers to seals during the short foraging migration (not pregnant)
TDR: Time-depth recorder
