# Supplementary material for: Summing the strokes: energy economy in northern elephant seals during large-scale foraging migrations
Source: Mov Ecol. 2015 Sep 15;3(1):22. doi: 10.1186/s40462-015-0049-2 (PMC4570705; doi:10.1186/s40462-015-0049-2)
Supplement: Additional file 1: Table S1. — Sensitivity results for calculations of field metabolic rate (FMR, in kJ kg-1 day-1) in adult, female northern elephant seals (Mirounga angustirostris) during the short and long foraging migrations. Table S2. Sensitivity results for calculations of locomotion costs (%Locom, as a percentage of ingested prey energy used) in adult, female northern elephant seals (Mirounga angustirostris) during the short and long foraging migrations. (DOCX 18 kb) [file 40462_2015_49_MOESM1_ESM.docx]

Table S.1. Sensitivity results for calculations of field metabolic rate (FMR, in kJ kg^-1^ day^-1^) in adult, female northern elephant seals (*Mirounga angustirostris*) during the short and long foraging migrations.

|  |  | Short Trip | | | |  | Long Trip | | | |
| --- | --- | --- | --- | --- | --- | --- | --- | --- | --- | --- |
| Values |  | CPS^a^ | Sn^b^ | %HIF^c^ | **FMR** |  | CPS^a^ | Sn^b^ | %HIF^c^ | **FMR** |
| Mean (± s.d.) |  | 2.58 | 2130708 | 0.116 | **95.4** |  | 2.58 | 4819889 | 0.116 | **71.6** |
|  |  |  |  |  | **(16.6)** |  |  |  |  | **(11.5)** |
| Lower Limit |  | 1.88 | 1691186 | 0.064 | **48.7** |  | 1.88 | 4078494 | 0.064 | **40.0** |
| Upper Limit |  | 3.78 | 2594603 | 0.180 | **170.3** |  | 3.78 | 5212870 | 0.180 | **114.9** |
| Contributions |  |  |  |  |  |  |  |  |  |  |
| 1st Partial Derivative |  | 28.5 | 3.7E-05 | 1.3E-02 |  |  | 21.2 | 1.2E-05 | 4.5E-03 |  |
| 2nd Partial Derivative |  | 1.5E-11 | 2.4E-23 | 1.4E-17 |  |  | -1.8E-12 | 2.5E-25 | -3.4E-18 |  |
| Variance Contribution |  | 77.1% | 18.9% | 4.1% |  |  | 88.3% | 9.5% | 2.2% |  |
| Mean Shift Contribution |  | 1.9E-12 | 4.6E-13 | 4.4E-13 |  |  | -2.3E-13 | 1.0E-14 | -2.4E-13 |  |

^a^Cost per stroke (J kg^-1^). Mean value, lower limit and upper limit values from [23] of main text.

^b^Total number of flipper strokes . Mean value, lower limit and upper limit values from data presented in main text (this study).

^c^Heat increment of feeding (% of metabolizable energy). Mean, lower limit and upper limit values from [37] of main text.

Table S.2. Sensitivity results for calculations of locomotion costs (%Locom, as a percentage of ingested prey energy used) in adult, female northern elephant seals (*Mirounga angustirostris*) during the short and long foraging migrations.

|  |  | Short Trip | | | | |  | Long Trip | | | | |
| --- | --- | --- | --- | --- | --- | --- | --- | --- | --- | --- | --- | --- |
| Values |  | %FeUr^a^ | %HIF^b^ | BMR-%K^c^ | %Work^d^ | **%Locom** |  | %FeUr^a^ | %HIF^b^ | BMR-%K^c^ | %Work^d^ | **%Locom** |
| Mean (± s.d.) |  | 0.170 | 0.116 | 1.0 | 0.686 | **0.102** |  | 0.170 | 0.116 | 1.0 | 0.661 | **-1.33E-04** |
|  |  |  |  |  |  | **(0.098)** |  |  |  |  |  | **(0.073)** |
| Lower Limit |  | 0.084 | 0.064 | 0.670 | 0.611 | **-0.098** |  | 0.084 | 0.064 | 0.670 | 0.627 | **-0.286** |
| Upper Limit |  | 0.217 | 0.180 | 1.10 | 0.888 | **0.273** |  | 0.217 | 0.180 | 1.10 | 0.694 | **0.229** |
| Contributions |  |  |  |  |  |  |  |  |  |  |  |  |
| 1st Partial Derivative |  | -1.0 | -1.0 | -0.381 | 1.0 |  |  | -1.0 | -1.0 | -0.468 | 1.0 |  |
| 2nd Partial Derivative |  | 2.37E-12 | -1.19E-11 | -1.21E-13 | 1.25E-13 |  |  | -2.01E-12 | 2.86E-12 | -6.24E-15 | -3.48E-14 |  |
| Variance Contribution |  | 15.4% | 1.8% | 23.3% | 59.6% |  |  | 27.3% | 3.1% | 62.6% | 6.9% |  |
| Mean Shift Contribution |  | 1.75E-12 | -1.01E-15 | -9.32E-16 | 3.59E-16 |  |  | -1.48E-15 | 2.42E-16 | -4.80E-17 | -6.48E-18 |  |

^a^Energy lost via feces and urine (% of gross energy ingested). Mean, lower limit and upper limit values from [25, 34, 35, 36] of main text.

^b^Heat increment of feeding (% of metabolizable energy). Mean, lower limit and upper limit values from [37] of main text.

^c^Basal metabolic rate (% of Kleiber predictions). Mean value, lower limit and upper limit values from [25, 42, 43, 70] of main text.

^d^Total energy not available for production (% of gross energy ingested). Mean value, lower limit and upper limit values from [23] of main text plus a,b,c above.
